# Examining the relationship between ssVEP and psychophysical measures of contrast sensitivity, grating acuity, and orientation discrimination

**DOI:** 10.1016/j.isci.2026.116063

**Published:** 2026-05-22

**Authors:** Martina Morea, Simona Garobbio, Marina Kunchulia, Michael H. Herzog

**Affiliations:** 1 Laboratory of Psychophysics, Brain Mind Institute, School of Life Sciences, École Polytechnique Fédérale de Lausanne (EPFL), 1015 Lausanne, Switzerland; 2 Research Institute of Cognitive Neurosciences, Free University of Tbilisi, Tbilisi 0159, Georgia

**Keywords:** neuroscience, physiology, sensory neuroscience

## Abstract

Intuitively, we categorize individuals as good or poor performers. In vision, someone who performs well on one test is expected to do well on other tests. If so, test results should correlate with each other. However, studies have found low correlations between basic visual tests. One possible explanation is the detrimental influence of non-visual factors—such as attention or motivation—on correlations. To minimize these effects, we adopted steady-state visually evoked potentials (ssVEPs), which do not rely on subjective responses. 35 healthy participants performed contrast sensitivity, visual acuity, and orientation discrimination tests with psychophysics and ssVEPs. Although both methods showed high reliability, correlations between tests were weak. Thus, tests might target more specific aspects of vision than normally assumed. Furthermore, psychophysical and ssVEP measures did not correlate, suggesting that test outcomes depend on the method rather than the targeted visual abilities or that ssVEPs and psychophysics have different systematic errors.

## Highlights

• Visual tests are assumed to reflect underlying mechanisms
• Behavioral tests of related visual abilities correlate only weakly with each other
• EEG tests of the same abilities also do not correlate with each other
• Tests might be more specific or idiosyncratic than assumed

## Graphical abstract

**Figure:**
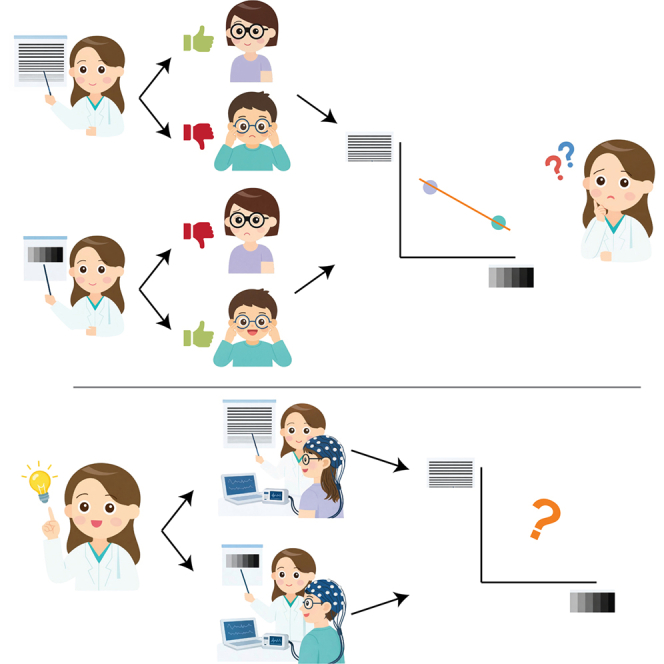

## Introduction

To assess a specific visual ability, such as contrast sensitivity, researchers typically use standard psychophysical tests. A common example is a contrast detection task, in which participants indicate the presence of a grating while its contrast is varied. Individuals who perform well on this task would be expected to also perform well on related tasks, such as an orientation discrimination task, based on the assumption that a common set of low-level visual mechanisms contributes to performance. For example, the density and distribution of photoreceptors in the retina, the tuning properties of neurons in the primary visual cortex (V1), and the efficiency of spatial frequency and contrast gain control processes. It follows that if an individual has a reduced density of photoreceptors, all tasks that rely on high spatial resolution will be affected. Thus, outcomes of tasks targeting the same construct (e.g., low-level vision) should correlate. However, research in the past decade has challenged this assumption. Studies in healthy young and older adults have reported only weak correlations between performances across basic visual tasks. For example, one study^1^ tested 40 participants with six basic visual tests, such as visual acuity, vernier discrimination, and contrast detection, and reported only weak correlations between pairs of tests, despite all tests showing high test-retest. Similar results were obtained by another study,^2^ with an extended battery of 19 visual tests, in both healthy young and older individuals. This suggests that performance in one test does not predict performance in another test (for reviews, see Mollon et al., Peterzell, and Tulver et al.^3^^,^^4^^,^^5^), suggesting that there is no obvious common factor underlying vision.

One possible explanation for these weak correlations is that psychophysical tests, while designed to target a certain visual mechanism, are influenced by non-visual factors such as attention, decision-making, and motor execution, which all may come with individual idiosyncrasies, i.e., a large individual variance.^6^ In addition, some tasks engage attention or motivation more strongly than others, thereby possibly reducing correlations.

The first goal of this study is to investigate whether correlations between tests increase when non-visual confounding factors are minimized. Steady-state visually evoked potentials (ssVEPs) provide a suitable approach, as they allow objective measurement of perceptual thresholds with minimal influence from behavioral or cognitive biases. In ssVEPs, stimuli are flickering with a certain frequency, which leads to oscillating brain responses of the same frequency and higher harmonics.^7^^,^^8^ High-contrast stimuli elicit higher response amplitudes than low-contrast ones; thus, a reduced oscillatory power can be interpreted as evidence that the brain is not processing or perceiving the stimulus. Hence, one can determine visual thresholds without requiring a response from the participants. For this reason, ssVEPs are widely used to estimate visual acuity and contrast sensitivity in both research and clinical contexts (for reviews, see Almoqbel et al., Hamilton et al., Norcia et al., and Zheng et al.^9^^,^^10^^,^^11^^,^^12^). Being independent of behavioral responses, this technique is well suited for pre-verbal children^13^^,^^14^^,^^15^^,^^16^^,^^17^ and patients with motor-learning or severe visual impairments.^18^^,^^19^^,^^20^^,^^21^^,^^22^^,^^23^

The second goal of the study was to assess correlations between psychophysical and ssVEP methods for the same visual ability. Studies have reported high test-retest reliability of electroencephalography (EEG)-based tests and significant correlations with psychophysical thresholds in clinical populations (for review, see Almoqbel et al.^9^) or when acuity is artificially reduced (see Figure 8 in Hamilton et al.^10^). The validity and reliability of this approach in such populations are therefore well established. However, results in healthy individuals are more mixed: while some studies report good correspondence between EEG-based and behavioral thresholds,^24^^,^^25^^,^^26^^,^^27^ others have failed to find a clear relationship.^28^^,^^29^^,^^30^ Here, we reexamined this question. If the low correlations observed between tests reflect the high specificity of these measures—that is, if they accurately and exclusively capture variations in contrast sensitivity or visual acuity—then EEG and behavioral tests designed to target the same ability should correlate even more strongly.

To this end, 35 healthy, young adults performed three ssVEP-based tests, estimating visual acuity, contrast sensitivity, and orientation discrimination, as well as the three corresponding psychophysical tests. Importantly, we focused on a healthy population to investigate basic visual processing under typical viewing conditions. Our aim was therefore to assess the validity of basic visual tests under normal conditions. While this approach necessarily limits the observable range, it allows us to probe variability within normal vision rather than differences driven by pathology or artificial manipulations.

To further increase our chances of finding correlations, the same sine-wave grating was used to measure contrast sensitivity, visual acuity, and orientation sensitivity for all tests. Because each stimulus in these tasks has a specific contrast, spatial frequency, and orientation—and since these visual abilities show mutual dependencies—we expected at least a common, stimulus-related factor.

## Results

We employed an ssVEP paradigm to estimate perceptual thresholds without requiring explicit behavioral responses from participants. EEG was recorded while participants passively viewed a stimulus presented at a rate of 20 pattern reversals per second. During stimulation, a single parameter was systematically varied over time—either contrast (Con), spatial frequency (SF), or orientation (Ori)—by gradually increasing or decreasing its intensity. Perceptual thresholds were then derived from the EEG signal by identifying the point at which the ssVEP response fell below a predefined signal-to-noise ratio (SNR) criterion. Among the range of available approaches, this method was selected as an operational definition of threshold appropriate for the present study (see the STAR Methods for further information).

### EEG responses

**Figure 1:**
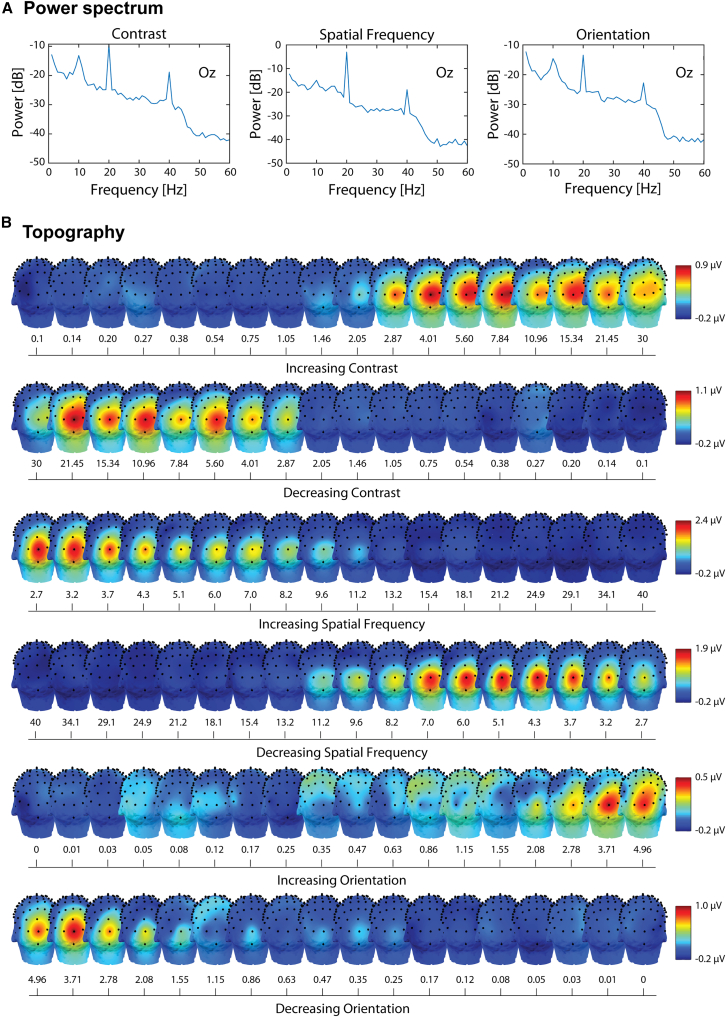
EEG responses (A) Mean power spectrum at electrode Oz after pooling increasing and decreasing trials across participants for each condition. Data re-referenced to the mediofrontal electrode, FPz. A peak is clearly visible in correspondence of the second (20 Hz) and fourth (40 Hz) harmonics. (B) Grand average across participants of topographical maps of the amplitudes adjusted for the noise floor, for each condition and each stimulus level. It is possible to see a gradual increase in activity across the stimulation sweep, with highest responses in correspondence of the highest stimulus intensities. The maximal responses are concentrated around the central occipital electrode, Oz.

To confirm that the stimulation elicited a strong brain response at the stimulation frequency, we plot the power spectrum of the signal at electrode Oz. As shown in Figure 1A, a clear peak is visible at the second harmonic (20 Hz) of the stimulation frequency. Additionally, a smaller yet prominent peak is present at the fourth harmonic (40 Hz). Figure 1B presents topographical maps of the amplitudes adjusted for the noise floor, averaged across participants. The maps illustrate a gradual increase in brain activity over the course of the stimulation sweep, with the first strong responses emerging near the central steps. As expected, brain activity related to the visual stimulus is strongly concentrated at the central occipital electrode Oz. Stimulation conditions for contrast and spatial frequency showed the strongest responses. However, a similar trend was observed for the orientation condition, though the signal appeared noisier.

**Figure 2:**
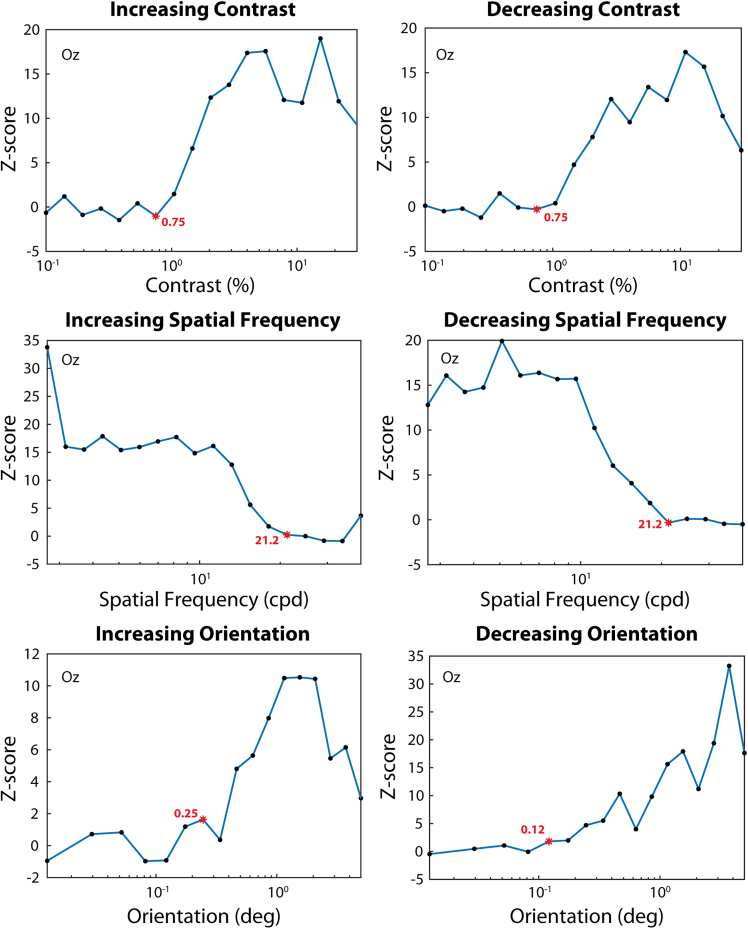
Threshold extraction *Z*-scored amplitudes for the six conditions, averaged across participants for illustration purposes. The name of the electrode, which was chosen for the analysis due to having the highest signal-to-noise ratio at the frequencies of interest, is shown in the plots (Oz). Graphs of the decreasing conditions are reversed for illustration purposes. The red asterisk indicates where the algorithm identified the threshold, with its value labeled in red.

To illustrate the threshold extraction procedure, Figure 2 shows the thresholds that would be selected for the grand average of all participants. Decreasing sequences are reversed to allow for direct comparisons with the increasing ones. The analysis is carried out on single electrodes, chosen with the signal-to-noise ratio criterion described in the STAR Methods section. For all conditions, the electrode Oz was selected as it produced the highest response at the frequencies of interest. The method detects meaningful and reliable thresholds, since they are in the expected performance range and nearly identical for both the increasing and decreasing conditions.

### Correlations

To assess the reliability of the test measures, we first computed intraclass correlations (see STAR Methods). We then used Spearman correlations to examine relationships within the psychophysical and EEG tests, as well as between the two.

#### Psychophysical data

**Figure 3:**
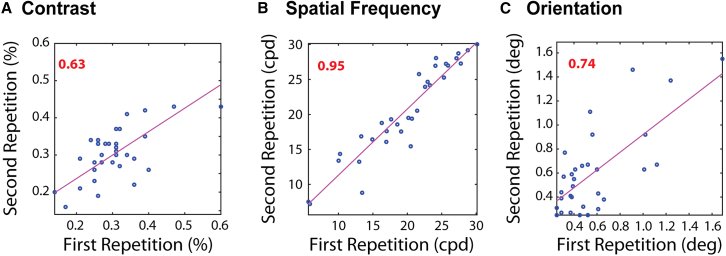
Test-retest reliability of the psychophysical measures Intraclass correlation (ICC) between the first and second measurement for (A) contrast, (B) spatial frequency, and (C) orientation is displayed. Each measurement took place in the same session. ICC values are shown in the top left. Red color stands for significant correlation (p<0.05). *N* = 35.

Test-retest reliability of the psychophysical dataset was medium-high (Figure 3): r=0.63, $p=3.06\times 10^{-5}$ for contrast, r=0.95, $p=1.44\times 10^{-15}$ for spatial frequency, and r=0.74, $p=5.22\times 10^{-7}$ for the orientation task. The tests used for contrast and orientation have been generally used in the past to measure such abilities,^1^^,^^31^ and here we confirmed their reliability

**Figure 4:**
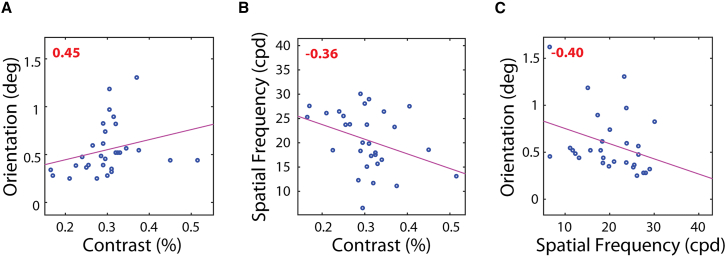
Correlations between the different visual tests for the psychophysical measures Spearman correlations between the different visual tests: (A) Ori vs. Con, (B) SF vs. Con and (C) Ori vs. SF. Spearman’s r is shown in the top left. Red color stands for significant correlations (p<0.05). Please note that for Con and Ori, better performance is indicated by a low score, whereas for spatial frequency, a higher score indicates better performance. *N* = 35.

Correlations between the psychophysical visual tests were medium and significant (Figure 4: orientation vs. contrast, r=0.45, p=0.01; spatial frequency vs. contrast, r=−0.36, p=0.05; and spatial frequency vs. orientation, r=−0.40,p=0.03).

The significant correlation between contrast and orientation was found before.^31^ The correlations between spatial frequency and the two other tests may stem from the use of a grating’s spatial frequency as a measure of visual acuity, in contrast to traditional methods that rely on letter or gap identification, such as the Snellen E, Landolt C, or vernier acuity.^1^

#### EEG

**Figure 5:**
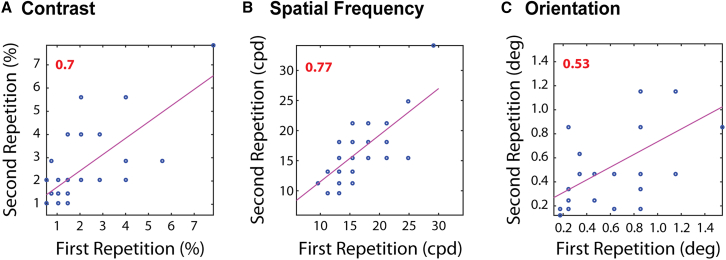
Test-retest reliability of the EEG measures Intraclass correlation (ICC) between the first and second measurement for (A) contrast, (B) spatial frequency, and (C) orientation is displayed, with scatterplots of variable pairs. Each measurement took place in the same session and is the average of thresholds for the increasing and decreasing sweeps. ICC values are shown in the top left. Red color stands for significant correlation (p<0.05). *N* = 35.

Test-retest reliability of the EEG dataset was high (Figure 5): r=0.70, $p=8.06\times 10^{-6}$ for contrast, r=0.77, $p=2.60\times 10^{-8}$ for spatial frequency, and r=0.53, p=0.004 for the orientation task.

**Figure 6:**
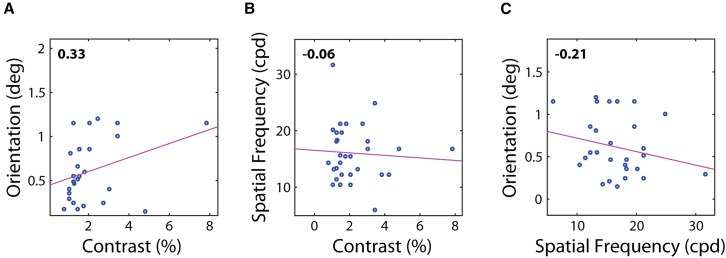
Correlations between the different visual tests for EEG-based thresholds Spearman correlations between the different visual tests: (A) Ori vs. Con, (B) SF vs. Con and (C) Ori vs, SF. Spearman’s r is shown in the top left. Red color stands for significant correlation (p<0.05). *N* = 35.

Correlations between the EEG thresholds are shown in Figure 6: all correlations were small and non-significant (orientation and contrast, r=0.33,p=0.10; spatial frequency and contrast, r=−0.06,p=0.73; and orientation and spatial frequency, r=−0.21,p=0.28).

#### Psychophysics vs. EEG

**Figure 7:**
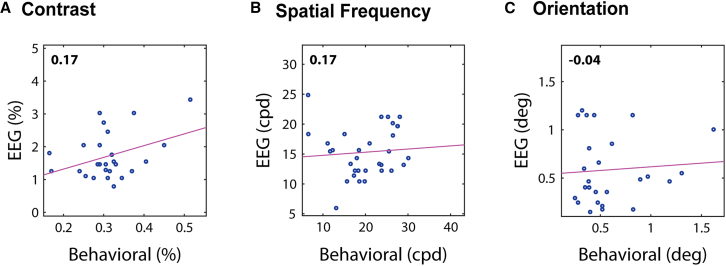
Correlations between psychophysical and EEG measures Spearman’s r is shown in the top left, none of which were found to be significant (p<0.05), neither for (A) contrast, (B) spatial frequency, nor (C) orientation. *N* = 35.

Since both psychophysical and EEG tests proved to yield reliable measures, one would expect to find a significant correlation between these two methods. However, no significant correlations were found, neither for contrast (Figure 7A, r=0.17,p=0.40), spatial frequency (Figure 7B, r=0.17,p=0.36), nor orientation (Figure 7C, r=−0.04,p=0.86).

## Discussion

The present study had two main goals. First, we aimed to determine whether a response-free method for assessing visual performance would increase between-test correlations by more selectively capturing underlying visual mechanisms. Second, we examined the relationship between EEG-based and behavioral threshold estimates. In both cases, we observed only weak correlations. These findings may reflect the engagement of partially distinct neural mechanisms across tests, as well as differences in the sources of systematic and/or statistical error both between and within methods. The following paragraphs explore these possibilities in more detail.

### Lack of between-test correlations

We found that the correlations between EEG-based thresholds were even smaller than the correlations between the psychophysical test. The initial hypothesis was that by removing non-visual aspects, unavoidable in behavioral tests, such as attention, motor execution, and response strategies, we would better isolate the underlying visual mechanisms, leading to stronger correlations between different visual abilities. The fact that the opposite pattern was observed leads to two main interpretations. First, if the confounding aspects are consistent across behavioral tasks (i.e., reflect participant traits), they may actually increase correlations. If that were the case, the higher between-test correlation observed in the psychophysical tests may reflect the influence of these confounding aspects rather than the underlying visual mechanism. This highlights the importance of assessing both test-retest reliability and test validity—specifically, how each test relates to other measures intended to probe similar processes—since reliance on a single test may offer only limited explanatory power. Multiple complementary tests may be necessary to fully characterize a given aspect of visual perception. Second, the lack of correlations between EEG-based thresholds suggests that each test may engage distinct visual mechanisms. Different stimuli are likely to activate different regions of the visual cortex, thereby eliciting different EEG responses. For instance, finer spatial frequency stimuli predominantly engage cortical representations of the central visual field, whereas coarser stimuli recruit a larger extent of the visual cortex.^32^^,^^33^^,^^34^^,^^35^ Such anatomical and physiological constraints are likely to have contributed to the observed variability in threshold estimates.

Previous work has demonstrated that multiple mechanisms underlie visual function by showing that the contrast sensitivity function is governed by several spatial frequency-tuned channels. In these studies, contrast thresholds are measured across multiple spatial frequencies and organized into pairwise correlation matrices, which are then subjected to factor analysis to estimate the number and tuning of latent variables, often interpreted as spatial frequency channels. This approach has also been applied to ssVEP data, revealing three distinct channels^36^ with tuning profiles closely matching those identified psychophysically^37^^,^^38^ and in masking experiments.^39^ Importantly, thresholds at low and high spatial frequencies were largely uncorrelated, while strong correlations emerged within frequency bands. These findings indicate that even within a single visual test, multiple independent mechanisms may contribute to performance.

Importantly, the absence of correlations in our dataset cannot be attributed to low variance in the data, often referred to as the reliability paradox.^40^ Variability within our sample was substantial for both the psychophysical and EEG datasets (spatial frequency: SD=6.28 and SD=4.91; orientation: SD=0.48 and SD=0.35, respectively), Moreover, observed ranges were comparable to those reported in previous studies involving young adult participants.^2^^,^^31^^,^^41^ The only exception was the contrast measure in the psychophysical dataset (SD=0.09; EEG dataset: SD=1.48), which showed a somewhat restricted range (0.17%–0.60%), likely due to the particular stimulus parameters that could have shifted contrast sensitivity ranges toward the lower stimulus intensity values. In particular, the relatively large stimulus size may have facilitated signal integration over broader areas of the visual field. Nevertheless, the test-retest reliability was good (ICC-r=0.63), confirming sufficient variance for correlational analyses.

### Lack of between-method correlations

Concerning the second goal of our study, no significant correlations were found between the thresholds derived from the two different assessment methods (psychophysics vs. EEG). This could be due to several factors, discussed in more detail in the following paragraph.

First, we observed overall higher thresholds (lower for visual acuity) in the EEG test results compared to the psychophysical ones (see Figure S6). It has been reported previously that EEG-based thresholds tend to be underestimated compared to behavioral thresholds.^10^^,^^42^^,^^43^^,^^44^^,^^45^ Electrodes being far from the sources, the type of signals they are able to capture (synchronous population activity), noise from other non-visual sources of processing in the brain, electric noise from amplifiers, and electromagnetic interference with external sources all lead to reduced sensitivity that might not allow assessment of small perceptual variations close to behavioral thresholds, especially for healthy, good-sighted individuals where thresholds are lower. Under these conditions, which are constrained by inherent anatomical and technical factors, increasing the amount of data does not necessarily lead to improved detectability. Thus, we chose not to extend data collection in order to remain consistent with the reference study^41^ and to keep a practical perspective where any approach intended for broader use should also remain time efficient and feasible. Hence, in our sample, EEG may primarily capture responses to high-intensity stimuli that generated a sufficiently strong signal-to-noise ratio,^27^ thereby limiting our ability to detect correlations across the full threshold range.

Another possible explanation for the difference in threshold ranges between the two methods is that behavioral and EEG-based thresholds reflect different visual mechanisms. This hypothesis has been proposed previously in studies comparing ssVEPs with a variety of behavioral measures (e.g., letter charts and Teller cards), primarily on the basis of differences in absolute threshold values.^9^^,^^10^^,^^46^ Electrophysiological measures might tap into different underlying neural mechanisms (e.g., less subcortical vs. cortical generators) than those measured by conscious perception in psychophysical tests; thus, it may not be meaningful to compare EEG-based and behavioral thresholds in absolute terms.^9^^,^^10^^,^^46^

We would like to highlight that in order to extract thresholds from the EEG, a fundamental modification has been made to the stimulus used in the behavioral tests: the flicker. This introduces potential confounds related to differences in processing at the neural population or cortical level. For example, the distinct band-pass properties of the magnocellular and parvocellular systems, which differ in their sensitivity to contrast and spatial frequency, might alter perception compared to psychophysical tests using static stimuli.^27^ High temporal frequencies (above 20 Hz) recruit mainly the magnocellular pathway.^47^^,^^48^^,^^49^^,^^50^ Thus, the pattern-reversal nature of the EEG stimulus may have engaged motion-sensitive pathways^51^^,^^52^ or induced different patterns of responses^53^^,^^54^ compared to motion-onset stimuli or the static stimulus used in the psychophysical task.

The choice of stimulation frequency can also influence the measured thresholds and thus acts as a confounding factor. In our study, in order to replicate previous results,^41^ the chosen stimulus frequency was close to alpha, a primary oscillatory component of visual processing that can sometimes exhibit harmonics,^55^ potentially contaminating the analyzed signal. Nonetheless, the power spectrum in Figure 1A reveals clear, distinct components at our frequencies of interest, indicating that this issue is unlikely to affect our results.

A further limitation of the EEG threshold extraction method used in this study might lie in its dependence on the choice of target and reference electrodes. We observed an improvement in signal quality—reflected in higher *Z*-scored amplitudes (see Figure S4) and test retest of contrast and orientation (Figure S5) when switching from the generally used common average reference to a reference composed exclusively of occipital electrodes (O1, O2, Oz, POz, and Pz). Similar effects of referencing strategies on EEG signal quality have been reported previously.^56^^,^^57^^,^^58^^,^^59^^,^^60^^,^^61^ This outcome may arise due to the nature of the noise sources subtracted during re-referencing. Specifically, when using a common average reference, noise originating from frontal and parietal regions—such as movement artifacts or muscle activity—that might present strong components despite being unrelated to visual processing can be introduced into the occipital signal. In contrast, referencing solely to electrodes close to the visual cortex ensures that the subtracted average noise is more localized to the occipital region, which is the primary area of interest for this visual task. Consequently, this approach enhances the signal-to-noise ratio, as the components being subtracted are more likely to represent true noise rather than task-irrelevant neural activity.

One more factor contributing to the observed lack of correspondence between behavior and EEG may be the inherent limitations in the threshold extraction approach used in this study, such as the significance method replicated in this study or the linear interpolation method,^10^^,^^12^ for identifying thresholds. Unlike psychophysical tests, for which the asymptotes of the psychometric function are well defined, EEG thresholds often rely on detecting the amplitude at a specific stimulation frequency exceeding a statistical threshold (the “noise floor”). While this standard significance-based criterion can reliably distinguish signal from noise, it may ignore other valuable information embedded in the amplitude response function, such as its shape or rate of change over different stimulus intensities. Furthermore, the inter-individual variability in the shape of the amplitude response function is large. Multiple factors, including electrode contact, scalp thickness, individual anatomy, and ambient noise,^62^^,^^63^ may contribute to the variability in EEG signal shape observed across participants. Thus, further work is necessary to optimize the algorithm in a way that provides reliable thresholds across the entire participant sample. Circular T^2^ statistics^64^ could improve detection by adapting the decision rule to include phase information. Spatial filtering^65^ and source separation techniques^66^ can capture richer information by integrating signals from multiple electrodes. Machine learning models have also already shown promise for improving correlations with psychophysical data.^67^

In conclusion, our study yielded two main findings. First, we found no evidence for a common visual factor when using an objective EEG-based measure of threshold detection across different visual functions. Although test-retest reliability was high, the different visual tests did not correlate with one another. This suggests that each test may tap into distinct aspects of visual processing. Therefore, it is essential to assess both test reliability (e.g., via test-retest measures) and test validity (e.g., by examining generalizability and relationships with other measures intended to probe the same mechanisms), as reliance on a single metric may provide an incomplete view of the underlying processes.

Second, we observed no correlation between EEG-based and behavioral threshold estimates in our sample. This discrepancy may arise from several confounding factors inherent to the EEG-based threshold detection method employed in this study: parameters such as stimulus design, task characteristics, and threshold extraction methods could all have influenced our results. Importantly, our results do not challenge the validity of the ssVEP method itself. We fully acknowledge its established reliability in clinical populations and in other contexts where it has been shown to provide robust and meaningful measurements. Nor do we suggest that ssVEP responses are unrelated to perception—on the contrary, perceptual processes necessarily arise from neural activity. Rather, our findings pertain specifically to the absence of statistically significant correlations within the present test battery, participant sample, and methodological framework. A lack of correlation in this context should not be interpreted as evidence of no underlying relationship. Future research is needed to better understand the extent to which confounding factors affecting both methods contributed to the lack of correlations observed here, as the relationship between ssVEP responses and perceptual thresholds remains an area of active discussion.

### Limitations of the study

The main limitation of this study is that, although the ssVEP paradigm we chose reduces confounds associated with behavioral feedback, it may introduce other sources of variability. These include hardware limitations, differences in the stimuli between the two methods (necessary to adopt an ssVEP paradigm), neural processing characteristics, and technical factors related to data preprocessing choices and threshold extraction algorithms. These factors may have influenced the correspondence between ssVEP-based and psychophysical measures. Nevertheless, previous studies employing comparable paradigms and analysis techniques have reported significant correlations between EEG and behavioral thresholds, suggesting that such correspondence can be achieved under appropriate conditions. Thus, further research is needed to establish the conditions under which this relationship holds and to clarify the link between ssVEP responses and perceptual thresholds.

## Resource availability

### Lead contact

Requests for further information and resources should be directed to and will be fulfilled by the lead contact, Martina Morea (martina.morea@epfl.ch).

### Materials availability

This study did not generate new unique reagents.

### Data and code availability

• Behavioral and EEG threshold data have been deposited at the Open Science Framework (OSF) and are publicly available as of the date of publication at https://doi.org/10.17605/OSF.IO/K5WRA.
• All original code has been deposited at the OSF and is publicly available at https://doi.org/10.17605/OSF.IO/K5WRA as of the date of publication.
• Any additional information required to reanalyze the data reported in this paper is available from the lead contact upon request.

## Acknowledgments

We would like to thank Marc Repnow for technical help in the study. M.M. was supported by the 10.13039/501100001711Swiss National Science Foundation (grant no. 310030L_212958, “The space in between: where local meets global in human vision”).

## Author contributions

Conceptualization, M.M., S.G., and M.H.H.; data curation, M.M. and S.G.; formal analysis, M.M. and S.G.; funding acquisition, M.H.H.; investigation, M.K.; methodology, M.M., S.G., and M.H.H.; software, M.M.; supervision, M.H.H.; visualization, M.M. and S.G.; writing – original draft, M.M., S.G., and M.H.H.; writing – review and editing, M.M., S.G., M.K., and M.H.H.

## Declaration of interests

The authors declare no competing interests.

## Declaration of generative AI and AI-assisted technologies in the writing process

During the preparation of this work, the authors used ChatGPT 5.3 in order to assist with language editing and spell checking. After using this tool/service, the authors reviewed and edited the content as needed and take full responsibility for the content of the published article.

## STAR★Methods

### Key resources table

**Table:** 

REAGENT or RESOURCE	SOURCE	IDENTIFIER
**Biological samples**		

EEG and psychophysical data	Laboratory of Psychophysics (LPSY) EPFL SV BMI	https://doi.org/10.17605/OSF.IO/K5WRA

**Software and algorithms**		

MATLAB	https://matlab.mathworks.com/	Version R2019b

**Other**		

Scripts for data processing	Open Science Framework (OSF) Repository	https://doi.org/10.17605/OSF.IO/K5WRA

### Experimental model and study participant details

Thirty-five young adults (21 females, $M_{age}=21.6$ years old, range 18–25) were university students recruited from Free University of Tbilisi, Georgia.

A description of participants’ demographics can be found in Table S1. Included participants had no history of epileptic seizures, were not at risk for epilepsy, or had any other neurological disorder. All had normal or corrected-to-normal vision, with a visual acuity of 1.0 (corresponding to 20/20) or better in at least one eye, as measured with the Freiburg Visual Acuity Test.^68^ Participants gave written informed consent prior to the experiment, were informed that they could withdraw from the experiment at any time, and were reimbursed for their participation.

The study adhered to the Declaration of Helsinki (except pre-registration) and was approved by the Independent Ethics Committee of the Beritashvili Center of Experimental Biomedicine in Tbilisi, Georgia (study approval number: #08/11072016).

### Method details

#### Psychophysical experiment

The stimuli were displayed on an ASUS VG248QE LCD monitor (53 cm × 30 cm, 1920 × 1080 pixels, 120 Hz). Participants were seated in a dimly illuminated room, at a 1 m distance from the screen. When applicable, they wore their glasses. For each test, a description of the task was provided and 10 practice trials were presented. An auditory feedback tone was provided after an incorrect response. After practice, tests were presented in randomized order and were repeated twice. All tests had 60 trials, except for the Freiburg Visual Acuity Test which had 30 trials.

Thresholds were determined using the QUEST adaptive procedure,^69^ aiming for a 75% correct response rate in a 2-alternative-forced-choice task, except for the Freiburg Visual Acuity Test, where the threshold was defined as 62.5% in a 4-alternative-forced-choice task.

The stimulus programs were implemented with the Psychophysics Toolbox^70^ and run on GNU Octave.^71^ Figure S1 shows a summary of the psychophysical tasks, and a description of each test is given below.

• Contrast (Con): the task was adapted from a previous study.^72^ Participants indicated whether a sine grating was oriented clockwise or counterclockwise with respect to the vertical axis of the screen (2-alternatives forced choice task). The grating had a mean luminance of 50 cd/m^2^, an orientation of 5°, a spatial frequency of 1.5 cycles per arcdeg (cpd), and a diameter of 15 arcdeg. The initial contrast value of the grating was 16% (Michelson). An annulus around the grating (luminance: 70 cd/m^2^) was added to signal the appearance of the stimulus. Dithering was used to improve the effective gray level resolution. Both the grating and the annulus were displayed for 200 ms. The next stimulus was presented 800 ms after the response. The contrast of the grating was changed adaptively over trials to measure the contrast threshold.
• Spatial frequency (SF): we used the same task and stimulus as in Con, with the parameter changing adaptively being the spatial frequency of the grating. The grating’s contrast was fixed at 30%. The initial spatial frequency was 1.5 cpd and was changed adaptively over trials to measure the upper spatial frequency threshold in cpd.
• Orientation (Ori): the same task and grating as for the two other tests (Con and SF) was used, but with fixed contrast (30%) and fixed spatial frequency (1.5 cpd). The degree of orientation of the grating with respect to vertical axis (orientation delta) was changed adaptively. Initial orientation delta was 5°. Minimum testable value was 0.25°. The orientation delta of the grating was changed adaptively to measure the orientation threshold in degrees.

#### EEG experiment

The same setup (screen, distance and room illumination) as in the psychophysical experiment was used for the EEG recordings. Data were gathered with a 64-electrodes Active Two Mk2 system (BioSemi B.V., The Netherlands) positioned with the standard 10/20 montage. Sampling rate was 2048 Hz.

For each ssVEP test, participants were instructed to fixate at a red dot in the center of the screen (4 arcmin diameter). At the end of every trial, participants had to report if the fixation dot changed color from red to green by pressing one of two handheld buttons. We included the task to ensure that participants attended to the screen throughout the whole duration of the stimulation.

The stimulus consisted of a vertical sinusoidal grating with the same diameter and luminance as the respective psychophysical experiment described in the previous section. We used a sweep VEP paradigm to measure visual acuity, contrast sensitivity and orientation sensitivity.

• Contrast: We used the same paradigm as in Hemptinne et al.^41^ The grating was presented with 20 pattern reversals per second (rps) to induce same-frequency responses in the brain (Figure S2A). Contrast was gradually increased or decreased throughout a trial over 18 logarithmically spaced steps (Figure S3A), between 0.1 and 30%. The spatial frequency and the orientation of the stimulus were kept constant at 1.5 cpd and 0° respectively.
• Spatial Frequency: Similar to contrast sensitivity, we used the same paradigm as in Hemptinne et al.^41^ Spatial frequency was varied over 18 logarithmically spaced steps between 2.7 and 40 cpd (Figure S3B). The contrast and the orientation of the stimulus were kept constant at 30% and 0°, respectively.
• Orientation: Flickering was obtained by changing the stimulus’ orientation, not the phase, between 0° and a positive angle, every 50 ms (Figure S2B). The tilt of the grating was varied over 18 logarithmically spaced steps between 0 and 4.96° with respect to vertical (Figure S3C). The contrast and spatial frequency of the stimulus were kept constant at 30% and 1.5 cpd, respectively. These parameter ranges were chosen based on previous research using comparable stimuli.^73^^,^^74^^,^^75^

To minimize luminance artifacts during the pattern reversal stimulus presentation, the monitor was set up for minimal overshoot/undershoot rather than for maximal transition speed. The stimulation lasted 1 s for each step. The first and last step of the sequences were repeated at the beginning and at the end of the sequence respectively. One trial lasted a total of 20 s (18 steps, 1s each, plus pre- and postview).

There were two possible sequence orders: increasing (from smaller amplitude of the parameter to larger) and decreasing (larger to smaller). We ran 6 trials for each sequence order and measure of interest (increasing/decreasing sequence of contrast/spatial frequency/orientation), for a total of 36 trials per participant. The order of the 6 conditions was randomized between participants. Every trial started with a gray screen and participants were asked to press a key to continue with the trial. After 2 s, the 20 s sequence started. The trial was concluded by 2 s of a gray screen. At the end of 6 trials (making up one block) there were 30 s of gray screen. The whole duration of the experiment, breaks included, was shorter than 30 min.

#### EEG analysis

##### Preprocessing

Data were pre-processed offline in MATLAB (R2019b, The MathWorks, Inc., Natick, MA), using the Chronux^76^ and EEGLAB^77^ toolboxes. The following steps were performed: down-sampling to 250 Hz, linear detrending, lowpass-filtering at 45 Hz, epoching (1s epochs, so the whole duration of one step), manual rejection of noisy channels and epochs by visual inspection (on average, 4% of trials were removed per participant), detection and removal of eye blink artifacts with Independent Component Analysis (ICA) and interpolation of bad channels. The clean EEG data was then re-referenced to the average of five occipital electrodes (O1, O2, Oz, POz, Iz). This approach is usually referred to as Laplacian filtering,^78^^,^^79^ in which local sources of activity are enhanced while reducing the contribution of distant noise sources that are likely unrelated to visual stimulation (see Figure S4).

##### Frequency analysis

We first conducted an exploratory analysis by plotting the periodogram after pooling increasing and decreasing trials for each condition, in order to assess the presence of peaks at the frequency of interest and potential harmonics. Electrode Oz was selected as a representative example. Then, epochs were averaged in the time domain after removing the preview and postview epochs. We followed the same procedure as.^41^ A Discrete Fourier Transform (DFT), with a frequency resolution of 1 Hz, was applied to each epoch. The 20 Hz frequency was chosen due to the flickering nature of the stimulus since the second harmonic carries the most of the signal for this type of stimulation and showed a large peak in the periodogram (Figure 1A). The amplitude of the transform at 20 Hz was adjusted for the noise floor by subtracting the average amplitude of the 12 neighboring frequency bins (6 by each side), skipping the immediately adjacent ones (19 and 21 Hz). Next, we plotted the amplitude adjusted by the noise floor across all electrodes for each condition and sweep direction. To do so, we averaged the four trials corresponding to each condition and sweep direction (Figure 1B). This allowed us to verify whether the activity exhibited a gradual increase or decrease, centered around the occipital electrodes.

For threshold extraction, we obtained a z-scored measure by dividing the resulted amplitude by the standard deviation of the 12 neighboring frequency bins.

##### Electrode selection

The threshold extraction was performed on single electrodes, selected based on their signal-to-noise ratio, for each participant and condition, as in Hemptinne et al.^41^ The best electrode was identified as the one with the highest *Z* score, averaged over the upper half of tested stimulus intensities. Specifically, this included the steps of 2.05-30% for contrast, 2.7-11.2 cpd for spatial frequency and 0.34-4.96° for orientation.

##### Threshold definition

In the psychophysical experiments, the threshold is defined as the steepest point in the psychometric function fitted as a cumulative Gaussian, corresponding to 75% correct responses for a 2-AFC task. However, this criterion does not directly apply to an EEG-based threshold definition, where the upper asymptote is not well defined, for various reasons (see discussion). Therefore, the EEG-based threshold was defined as the lowest stimulus intensity at which there was a significantly high amplitude in the EEG response. This approach allows to consider only the lower asymptote of the response curve for threshold definition. To this end, as in Hemptinne et al.,^41^ a significance criterion was applied to the z-scored signal at the trial-average level (z>2.33), providing a significance value of 0.01. For a sequence with decreasing stimulus intensity (i.e., contrast decreasing, spatial frequency increasing, or orientation decreasing), the threshold was selected as the last non-significant step which had at least three significant steps out of the four preceding steps. For a sequence with increasing intensity, it was the opposite. We refer to this method as the “Sig method” throughout the article.

To enable comparison with state-of-the-art methods, we also implemented an alternative approach (for a review, see Table 1 in Zheng et al.^12^), the linear extrapolation method, which we refer to as the “Reg method”. In this method, a linear regression is fitted to the raw amplitude at the frequency of interest as a function of either linear spatial frequency or log contrast.^26^^,^^44^^,^^80^^,^^81^^,^^82^^,^^83^^,^^84^ The fit is applied between the first peak with a signal-to-noise ratio (SNR) of at least 3—where SNR is defined as the amplitude divided by the average noise floor—and the point at which the curve drops below the average noise floor. The threshold is then defined as the zero-crossing of the fitted line. In order for the threshold to be valid, the phase must be constant or gradually leading (for contrast trials) or lagging (for spatial frequency trials) the stimulus. For a detailed description of the algorithm, see previous studies.^44^^,^^83^^,^^85^ For the orientation test, which was not used by previous studies,^83^^,^^85^ we considered a threshold to be valid if the phase remained constant. As the Reg method produced results comparable to those of the Sig method, we present only the latter in the following sections. Results from the Reg method are provided in the supplementary material (Figures S7 and S8).

### Quantification and statistical analysis

#### Outliers removal

An outlier removal procedure was applied to the raw psychophysical and the EEG thresholds to remove results of participants who displayed exceptional performance instability across the two correlated measures. We calculated the absolute score differences (*diff*, i.e., the absolute value of the score difference between the two test repetitions/two tests) and then z-scored the obtained values as follows:

Zdiff=0.6745∗(diff-median(diff))/(median(diff-median(diff)))

Measurements that gave a *Zdiff* larger than 3 were excluded.^86^ Between repetitions of the same test, 3 test results out of 105 (35participants·3tests) were excluded from the EEG dataset, 8 test results out of 105 were excluded from the Psychophysical dataset. In Psychophysics vs EEG, 5 test results out of 105 were excluded from both datasets. Test results and entries removed through outlier detection can be found in Tables S2 and S3.

#### Test-retest reliability

In the EEG dataset, test-retest reliability was assessed by computing two-way mixed effects models, i.e., intraclass correlation of type (3,1), ICC31,^87^^,^^88^ between the increasing and decreasing trials for each condition. In the psychophysical dataset, ICC was computed between the two repetitions for each condition (e.g., contrast sensitivity repetition 1 vs. contrast sensitivity repetition 2). The results of this analysis can be found in the correlations section.

#### Between test correlations

To compute correlations between the different visual tests, we averaged the valid scores of the two repetitions to obtain one score for each participant for each test.

These average scores from the two test repetitions were used to compute Spearman’s rank correlations within a dataset (e.g., in the psychophysical dataset, we computed Spearman’s rank correlations between contrast and visual acuity, contrast and orientation, and visual acuity and orientation).

To interpret the strength of correlations, we used Cohen’s guidelines,^89^ which consider correlation coefficients of 0.1, 0.3 and 0.5 as small, medium and large in magnitude, respectively. The results of this analysis can be found in the correlations section.

#### Between method correlations (psychophysics vs. EEG)

We followed the same procedure as described in the previous paragraph, but this time applied it between the two methods (Psychophysics vs EEG). The results of this analysis can be found in the correlations section.
