# Supplementary material for: Examining the relationship between ssVEP and psychophysical measures of contrast sensitivity, grating acuity, and orientation discrimination
Source: iScience. 2026 May 22;29(6):116063. doi: 10.1016/j.isci.2026.116063 (PMC13223984; doi:10.1016/j.isci.2026.116063)
Supplement: Document S1. Figures S1–S8 and Tables S1–S3 [file mmc1.pdf]

iScience, Volume *29*

Supplemental information

Examining the relationship between ssVEP and psychophysical measures of contrast sensitivity, grating acuity, and orientation discrimination

Martina Morea, Simona Garobbio, Marina Kunchulia, and Michael H. Herzog

Supplementary material

ID	*Age*	*Gender*	*Glasses*	*General health problems*
P1	24	F	Contact Lenses	No
P10	22	F	Yes	No
P11	22	F	Yes	No
P12	24	F	No	No
P13	24	M	No	No
P14	20	M	No	No
P15	22	F	No	No
P16	24	F	No	No
P17	22	F	No	No
P18	22	F	Yes	No
P19	22	M	No	No
P2	22	F	No	No
P20	22	F	No	No
P21	19	M	No	No
P22	23	F	No	No
P23	19	M	No	No
P24	22	M	No	No
P25	22	M	Yes	No
P26	22	F	No	No
P27	22	M	No	No
P28	25	F	No	No
P29	21	F	No	No
P3	19	M	No	No
P30	21	F	No	No
P31	21	M	Contact Lenses	No
P32	21	F	No	No
P33	21	F	Yes	No
P34	21	M	No	No
P35	20	F	No	No
P4	18	M	No	No
P5	19	M	No	No
P6	19	M	No	No
P7	22	F	No	No
P8	22	F	No	No
P9	22	F	Yes	No

Supplementary Table 1: Participants demographics. Related to STAR Methods.

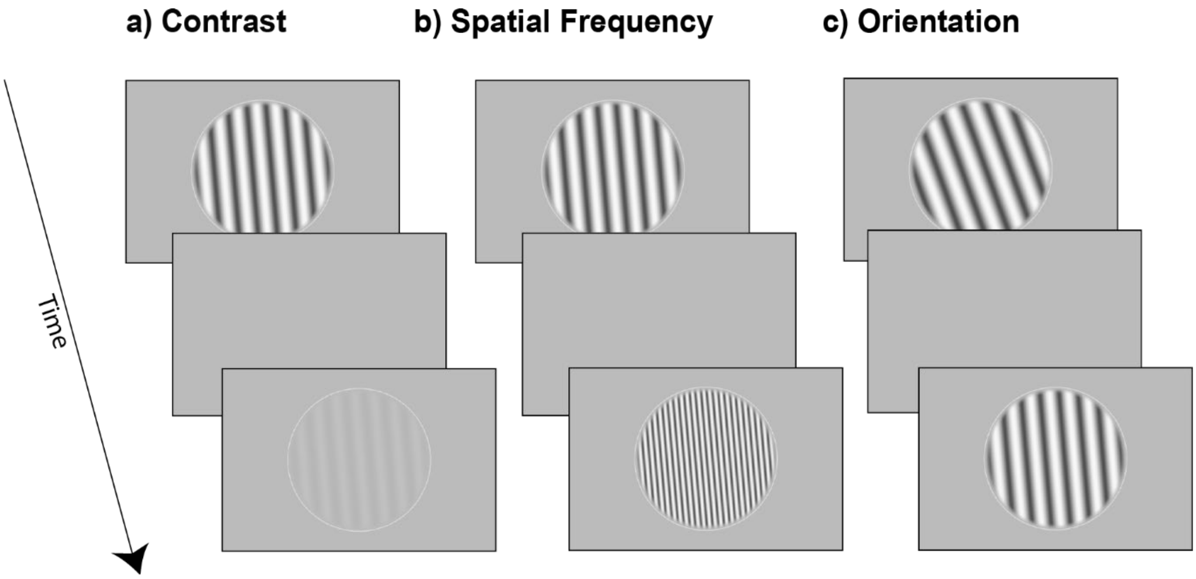

Supplementary Figure 1: Psychophysical tests. Related to STAR Methods. (a) Contrast; (b) Spatial Frequency; (c) Orientation. Observers judged the orientation of the grating in a 2-Alternatives Forced Choice task. The task becomes progressively harder or easier (by manipulating the parameter of interest) depending on the participant's performance. The stimuli's contrast, spatial frequency and orientation have been adapted for illustration purposes.

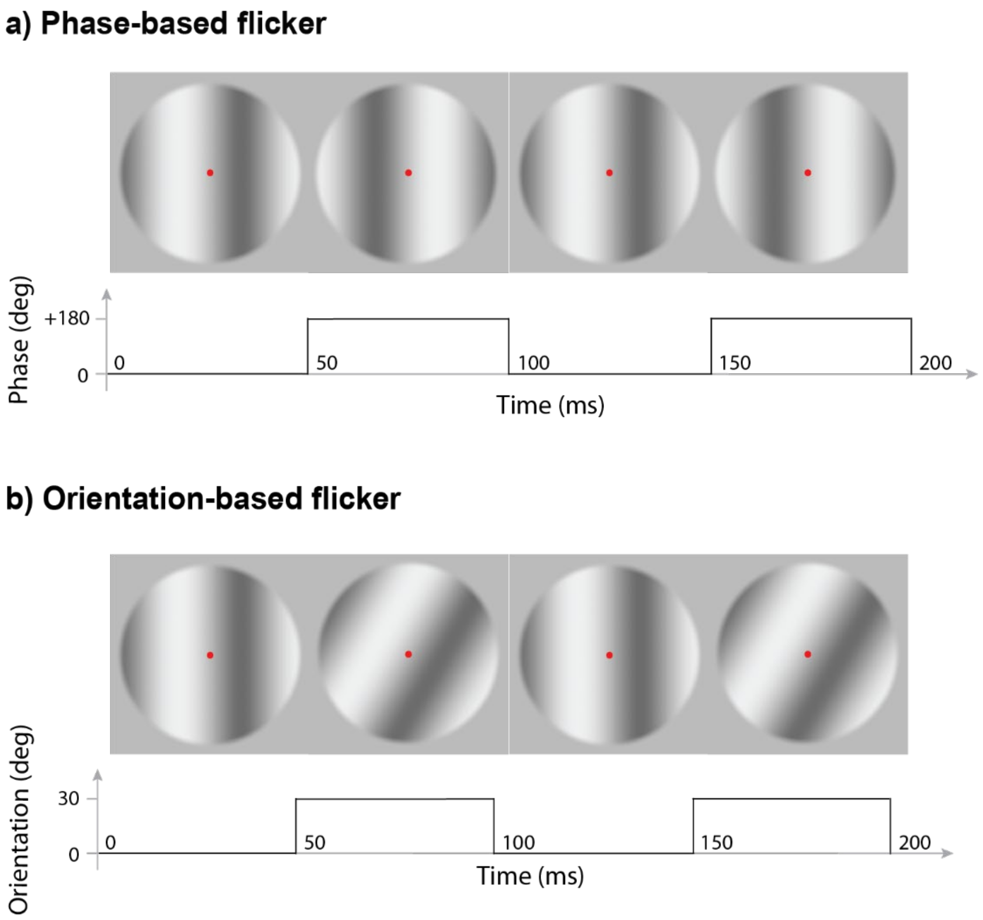

Supplementary Figure 2: flickering of the grating. Related to STAR Methods. The stimulus either pattern-reversed (i.e., switched phase from 0° to 180°) as in (a) or changed orientation with respect to vertical (0°), 30° in this example (b). The change occurred every 50 ms (according to 20 rps). The stimuli's contrast, spatial frequency and orientation together with the size of the fixation dot have been adapted for illustration purposes.

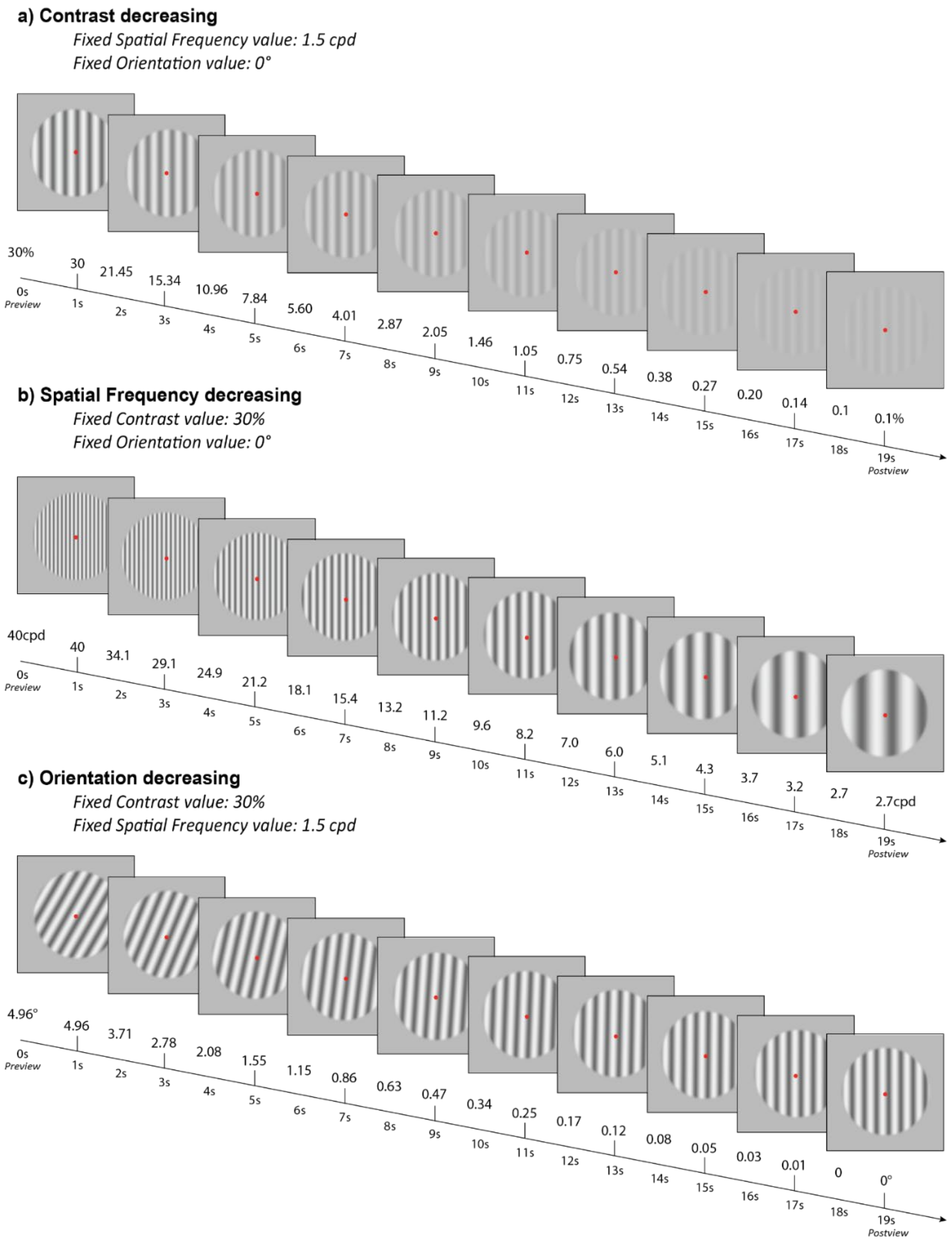

Supplementary Figure 3: EEG experiment. Three conditions out of the 6 tested are shown. Related to STAR Methods. Example of one trial (one sweep sequence) for (a) Contrast decreasing, (b) Spatial Frequency decreasing and (c) Orientation decreasing. A sine grating was shown at the center of the screen together with a fixation dot. The parameter of interest was gradually modulated over 18 logarithmically spaced steps. An additional step of one second was added at the beginning (preview) and the end (postview) of the sequence. The grating flickered at 20 reversals per second (rps) in (a) and (b) and through orientation change in (c).

ID	*Contrast increasing*	*Contrast decreasing*	*Spatial Frequency increasing*	*Spatial Frequency decreasing*	*Orientation increasing*	*Orientation decreasing*
P1	*7.84*	*7.84*	15.45	18.10	1.15	1.15
P10	1.05	1.46	18.10	21.21		*1.15*
P11	0.54	1.05	13.18	15.45	0.17	0.17
P12	0.54	2.05	15.45	21.21	0.47	0.47
P13	2.05	4.01	15.45	18.10	**0.34**	**1.55**
P14		2.05	9.60	11.25		
P15	2.05	2.05	13.18	11.25	0.86	0.86
P16			15.45	11.25	1.15	
P17	1.05	1.05	29.13	34.13	0.25	0.34
P18	1.46	1.05	15.45	11.25	0.25	0.86
P19	2.05	2.05	15.45	15.45		1.15
P2	**0.75**	**5.60**	21.21	15.45	0.47	0.25
P20	4.01	2.87	24.86	24.86	0.86	1.15
P21	1.46	4.01	21.21	21.21	0.25	0.25
P22	1.46	1.46	15.45	13.18		0.17
P23	*2.05*	*5.60*	11.25	13.18		
P24	1.05	1.46	13.18	9.60	0.34	0.63
P25	2.87	4.01	5.97			1.15
P26	1.46	2.05	15.45	15.45	0.25	0.17
P27	1.46	1.46	13.18	18.10	0.86	0.47
P28	1.05	1.46	18.10	18.10	0.25	0.25
P29	4.01	5.60	18.10	15.45	0.17	0.12
P3	1.46	1.46	21.21	21.21	0.86	0.17
P30	1.46		9.60	11.25		
P31	2.87	2.05	13.18	13.18	*1.55*	*0.86*
P32	*5.60*	*2.87*	13.18	11.25		
P33	0.75	2.87	21.21	21.21	0.86	0.34
P34	1.05	2.05	13.18	11.25	0.63	0.47
P35			11.25	13.18		
P4	0.75	1.46	13.18	13.18	1.15	0.47
P5	1.05	2.05	21.21	18.10	0.86	
P6	1.05	1.05	24.86	15.45	0.47	0.25
P7	1.05	1.05	11.25	9.60	0.63	0.17
P8	**0.75**	**7.84**	13.18	18.10	0.47	0.47
P9	4.01	2.05	18.10	18.10	0.34	0.47

Supplementary Table 2: Significance method EEG thresholds extraction and measurements excluded through outlier's detection. Related to STAR Methods. Entries in bold were removed with the z-score-based outliers detection method for test-retest, in italic for between methods. Empty cells indicate non-definable thresholds (no existing datapoint satisfying the threshold definition criterion).

ID	*Contrast increasing*	*Contrast decreasing*	*Spatial Frequency increasing*	*Spatial Frequency decreasing*	*Orientation increasing*	*Orientation decreasing*
P1	*0.31*	*0.33*	**27.54**	**21.13**	1.01	0.63
P10	0.14	0.2	27.14	28.04	*0.29*	*0.27*
P11	0.36	0.29	20.5	15.44	0.66	0.38
P12	0.31	0.3	13.31	16.86	0.91	1.46
P13	0.34	0.41	13.44	8.79	0.32	0.77
P14	0.47	0.43	18.55	18.57	0.31	0.57
P15	0.3	0.28	19.1	17.55	0.6	0.63
P16	0.31	0.37	16.95	16.07	**0.9**	**3.11**
P17	0.27	0.28	**16.85**	**26.74**	0.25	0.25
P18	0.39	0.35	22.58	23.94	1.24	1.37
P19	0.21	0.29	24.08	26.96	0.29	0.44
P2	0.32	0.27	5.64	7.5	0.61	0.3
P20	**0.39**	**0.8**	5.85	7.19	1.69	1.55
P21	0.26	0.34	27.42	28.73	0.25	0.31
P22	0.36	0.22	30.14	30.03	0.54	1.11
P23	*0.26*	*0.33*	20.67	19.39	0.56	0.92
P24	0.31	0.32	17.00	17.6	1.12	0.67
P25	0.6	0.43	13.01	13.23	0.39	0.49
P26	0.34	0.3	10.02	13.38	0.41	0.63
P27	0.27	0.3	10.23	14.35	0.38	0.59
P28	0.25	0.23	25.91	27	0.4	0.55
P29	**0.41**	**0.63**	21.43	20.53	0.48	0.32
P3	0.28	0.33	21.7	25.77	1.02	0.92
P30	0.4	0.26	14.92	16.42	0.61	0.43
P31	0.31	0.31	28.79	29.18	*0.37*	*0.27*
P32	*0.25*	*0.28*	22.95	24.66	0.52	0.67
P33	0.17	0.16	25.32	25.27	0.29	0.39
P34	0.36	0.29	16.29	18.78	**3.45**	**0.68**
P35	0.21	0.21	24.17	28.06	0.25	0.25
P4	0.25	0.26	23.28	24.17	0.38	0.4
P5	0.39	0.42	27.77	27.28	**2.21**	**0.85**
P6	0.32	0.37	25.62	27.24	0.47	0.66
P7	0.29	0.33	20.16	19.5	0.45	0.25
P8	0.26	0.19	17.65	19.3	0.52	0.25
P9	0.24	0.34	**29.72**	**22.04**	0.37	0.41

Supplementary Table 3: psychophysical threshold and measurements excluded through outlier's detection. Related to STAR Methods. Entries in bold were removed with the z-score-based outliers detection method for test-retest, in italic for between methods.

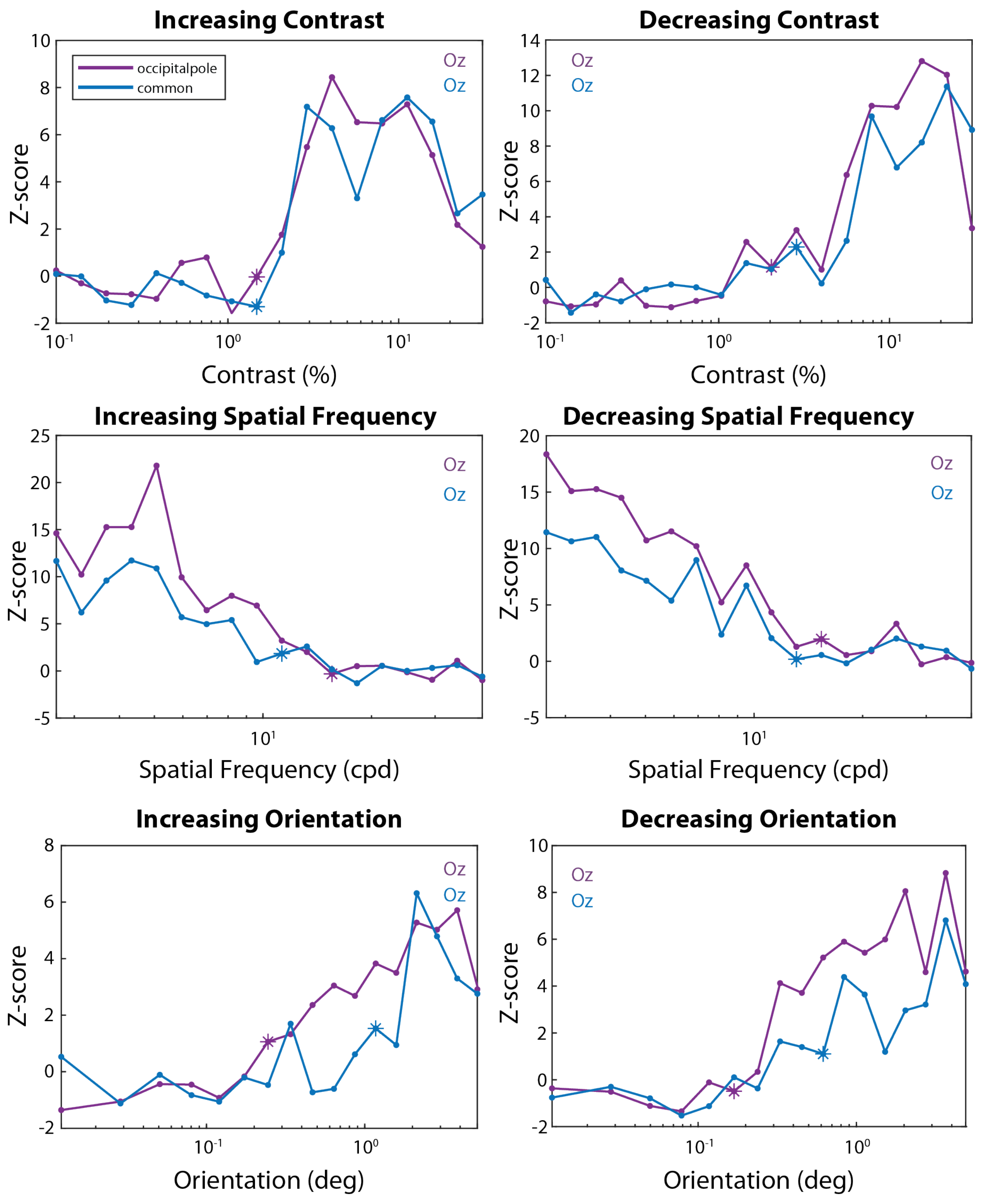

Supplementary Figure 4: Difference between common average re-reference and occipital re-reference for one representative subject. Related to Discussion. Z-scored amplitudes for the six conditions, at the best electrode (Oz for both re-reference types and all conditions). The purple curve corresponds to occipital re-referencing, the blue curve is common average re-referencing. Graphs of the decreasing conditions are reversed for illustration purposes. The asterisks indicate where the algorithm identified the threshold. Occipital re-referencing yielded overall better results, with higher z-scores, lower thresholds and more stable responses.

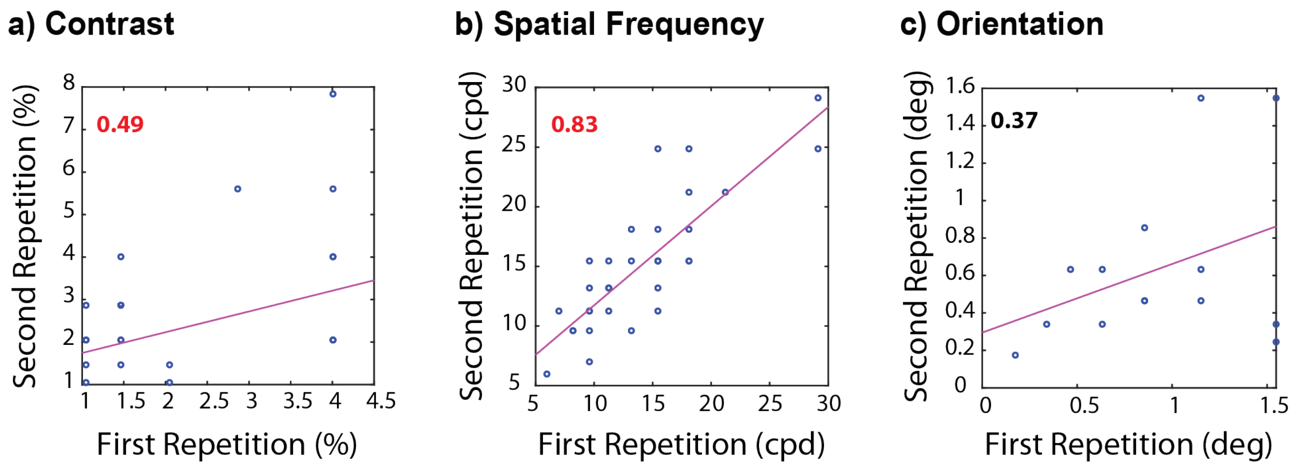

Supplementary Figure 5: Test-retest reliability of the Sig method's EEG measures with common average re-referencing. Related to Discussion. Intraclass correlation between the first and second measurement for (a) Contrast, (b) Spatial Frequency and (c) Orientation are displayed, with scatter plots of variable pairs. ICCs values are shown in the top left. Red color stands for significant correlation ($\boldsymbol{p < 0.05}$). The common average choice of re-referencing gives a lower reliability for contrast and orientation compared to the occipital re-referencing, with fewer participants for which it was possible to define a threshold in both increasing and decreasing trials.

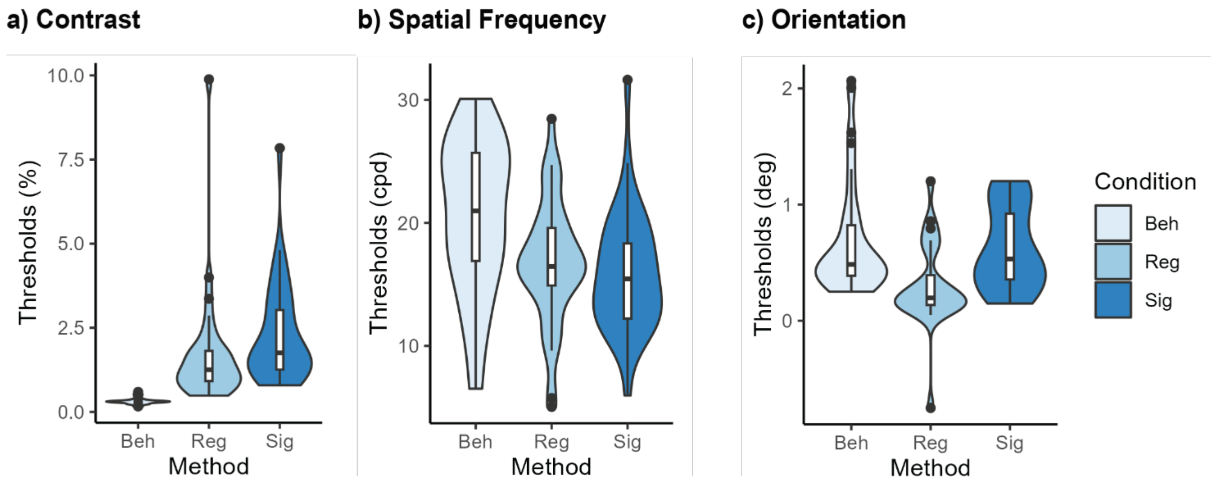

Supplementary Figure 6: Distribution of the thresholds for the three Behavioral tasks and the Sig and Reg method. Related to Discussion. The violin plots show the kernel probability density of the various Psychophysical and EEG measures. The horizontal line is the median, the extreme of the boxplots are the interquartile ranges. EEG thresholds are generally underestimated compared to Psychophysical thresholds.

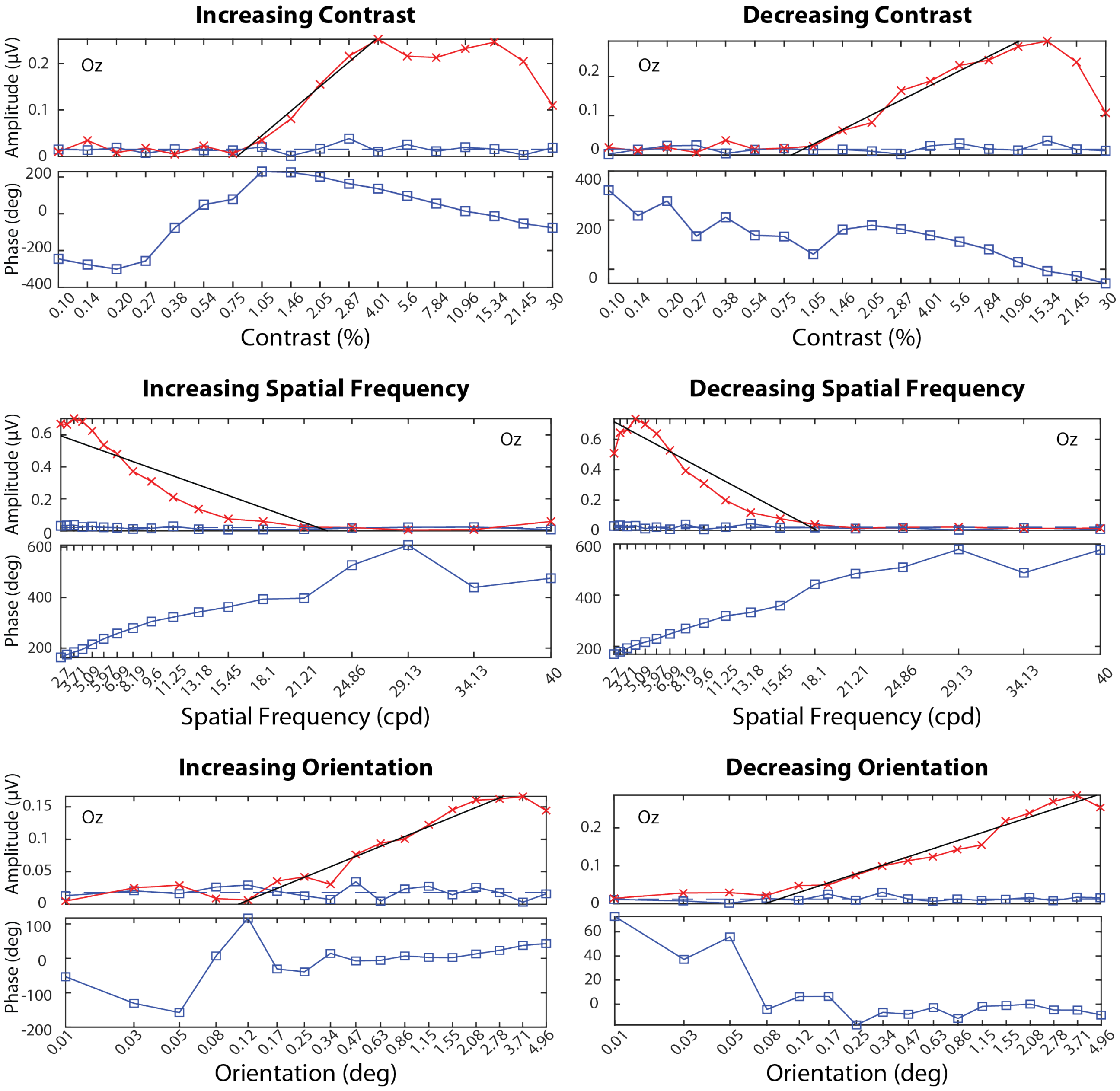

Supplementary Figure 7: Threshold extraction with the Regression method for the pooled dataset of all participants. Related to STAR Methods. Top graphs: Amplitudes at 20 Hz for the six conditions in red, noise amplitude i.e. at 22 Hz in blue. The dashed blue line is the average of the noise amplitude. The black line indicates the linear fit. The threshold is selected as the zero-crossing of the black line. Bottom graph: phase at 20 Hz. Data is shown at the Oz electrode. Graphs of the decreasing conditions are reversed for illustration purposes.

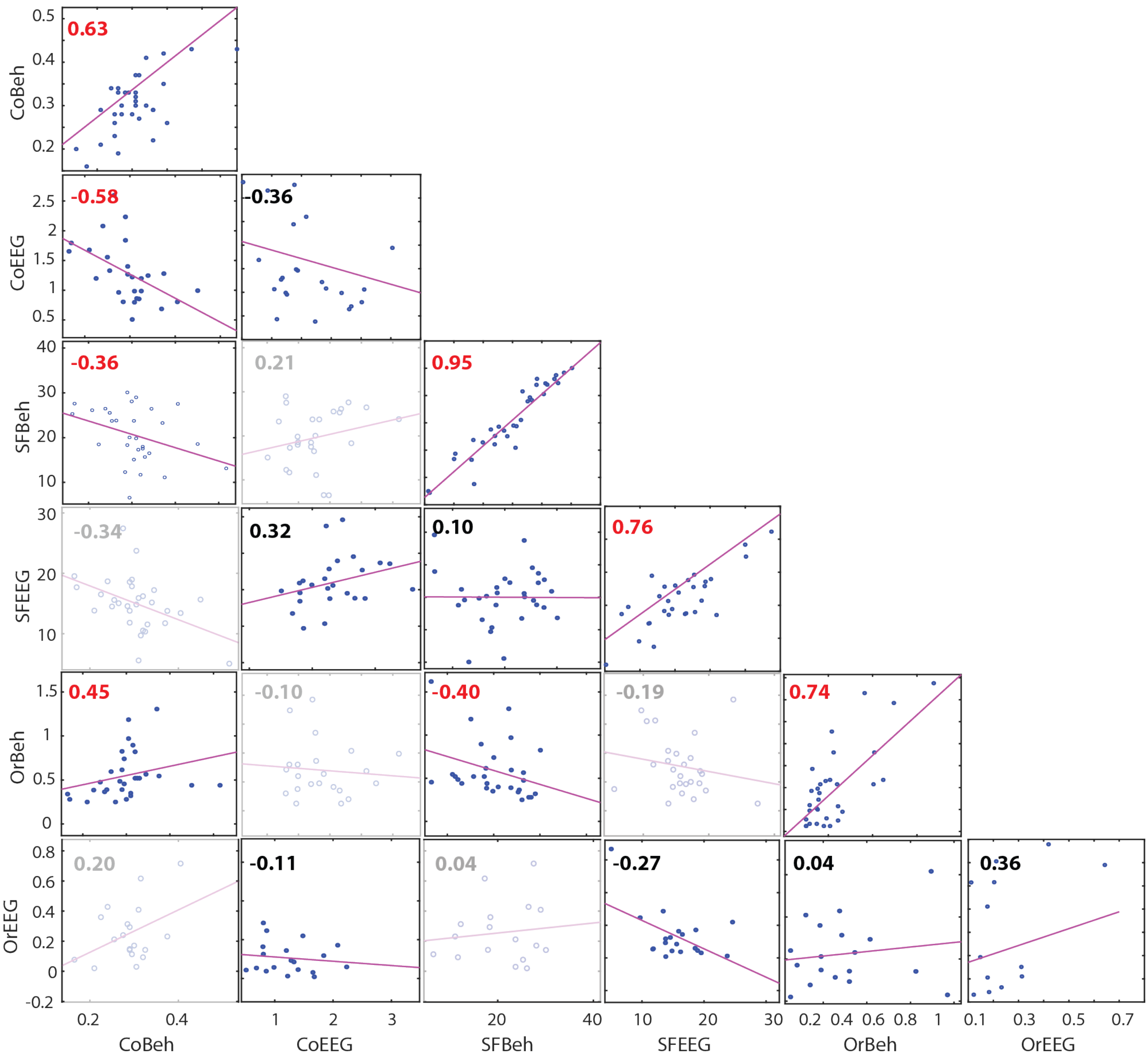

Supplementary Figure 8: Correlations for the Regression method. Related to STAR Methods. Spearman's-r is shown in the top left of each correlation plot. Red color stands for significant correlation ($\boldsymbol{p < 0.05}$). Diagonals report the ICC-r for test-retest reliability. Test-retest reliability was high for Spatial Frequency only. For Contrast and Orientation, the threshold extraction succeeded in both increasing and decreasing conditions for 27 and 16 out of 35 participants respectively. Furthermore, no correlations with the psychophysical measures were found
